# hnRNP A1B, a Splice Variant of *HNRNPA1*, Is Spatially and Temporally Regulated

**DOI:** 10.3389/fnins.2021.724307

**Published:** 2021-09-24

**Authors:** Myriam Gagné, Jade-Emmanuelle Deshaies, Hadjara Sidibé, Yousri Benchaar, Danielle Arbour, Alicia Dubinski, Gurleen Litt, Sarah Peyrard, Richard Robitaille, Chantelle F. Sephton, Christine Vande Velde

**Affiliations:** ^1^Department of Biochemistry, Université de Montréal, Montréal, QC, Canada; ^2^Centre de Recherche du Centre Hospitalier de l’Université de Montréal (CRCHUM), Montréal, QC, Canada; ^3^Department of Neurosciences, Université de Montréal, Montréal, QC, Canada; ^4^Department of Psychiatry and Neuroscience, CERVO Brain Research Centre, Laval University, Quebec City, QC, Canada

**Keywords:** RNA binding protein, central nervous system, motor neuron, amyotrophic lateral sclerosis (ALS), hnRNP

## Abstract

RNA binding proteins (RBPs) play a key role in cellular growth, homoeostasis and survival and are tightly regulated. A deep understanding of their spatiotemporal regulation is needed to understand their contribution to physiology and pathology. Here, we have characterized the spatiotemporal expression pattern of hnRNP A1 and its splice variant hnRNP A1B in mice. We have found that hnRNP A1B expression is more restricted to the CNS compared to hnRNP A1, and that it can form an SDS-resistant dimer in the CNS. Also, hnRNP A1B expression becomes progressively restricted to motor neurons in the ventral horn of the spinal cord, compared to hnRNP A1 which is more broadly expressed. We also demonstrate that hnRNP A1B is present in neuronal processes, while hnRNP A1 is absent. This finding supports a hypothesis that hnRNP A1B may have a cytosolic function in neurons that is not shared with hnRNP A1. Our results demonstrate that both isoforms are differentially expressed across tissues and have distinct localization profiles, suggesting that the two isoforms may have specific subcellular functions that can uniquely contribute to disease progression.

## Introduction

The heterogeneous ribonucleoprotein (hnRNP) family is composed of multiple proteins regrouped into 13 families. hnRNP family diversity derives from successive gene duplication events and family members that are more closely related among higher eukaryotes permits diversification of underlying phenotypic differences (Barbosa-Morais et al., 2012; Busch and Hertel, 2012). hnRNPs are characterized by their ability to bind RNA and DNA via their RNA binding domains (RBDs). The function of different hnRNP family members varies by the specificity of their RNA binding properties, the composition of their different domains, and their subcellular localization (Krecic and Swanson, 1999; Han et al., 2010b; Geuens et al., 2016; Bampton et al., 2020). Most hnRNP family members contain a nuclear localization sequence (NLS) which mediates their nucleocytoplasmic shuttling. At steady state, hnRNPs are predominantly localized to the nucleus, but are translocated to the cytoplasm following different stimuli or via their recruitment by other RBPs. hnRNPs are also enriched in intrinsically disordered regions (IDRs) which mediate protein-protein interactions and facilitate liquid-liquid phase separation (LLPS), a key event that governs the formation of biomolecular condensates (Geuens et al., 2016; Bampton et al., 2020; Milkovic et al., 2020).

RNA binding proteins (RBPs) are critical to all cellular processes and their expression is tightly regulated during cellular differentiation, stress response and aging (Kishore et al., 2010). The misregulation of RBP expression and function is often linked to disease (Lukong et al., 2008; Mittal et al., 2009; Ramakrishnan and Janga, 2019). A further complexity is that many RBPs can be alternatively spliced to generate conserved isoforms having unique functions or localization, even though they share common features (Krecic and Swanson, 1999; Xu et al., 2001, 2019; Sarkar et al., 2003; Liu et al., 2008; Han et al., 2010a,b; Gueroussov et al., 2017). For example, the inclusion of one exon in PTBP1 or hnRNP D modifies their efficiency to alternatively splice their targets (Gueroussov et al., 2015, 2017). Interestingly, most of the alternative splicing events observed in hnRNPs are located in their IDR, and these events are usually mammalian-specific. For example, hnRNP A and D subfamilies contain mammalian-specific alternative exons within their IDRs that are enriched for glycine-tyrosine (GY) in the C-terminus. Those isoforms have different impacts on alternative splicing and have altered phase separation properties (Gueroussov et al., 2017). Thus, alterations in RBP isoform usage can impact multiple targets. Moreover, mutations in the IDRs of hnRNP family members are associated with several diseases and frequently result in protein mislocalization and aggregation (Kim et al., 2013; Conlon and Manley, 2017; Purice and Taylor, 2018; Bolognesi et al., 2019; Gui et al., 2019; Batlle et al., 2020). However, studies of RBPs typically focus on only one isoform and the differential expression and regulation of RBP isoforms is often overlooked. Thus, understanding the function of these alternative isoforms will inform how their misregulation may contribute to different disease phenotypes.

As an RBP, hnRNP A1 contains two RNA recognition motifs (RRM1 and RMM2, collectively referred to as the UP1 domain) each composed of two RBDs, and an RGG box which features repetition of RGG which serves to mediate nucleic acid binding while promoting phase separation. In addition, hnRNP A1 has IDRs that are enriched in glycine (glycine-rich domain; GRD) and mediate protein-protein interactions and phase separation, and a C-terminal M9 sequence which determines its nucleocytoplasmic shuttling (Siomi and Dreyfuss, 1995; Ding et al., 1999; Ghosh and Singh, 2020; Martin et al., 2021). *HNRNPA1* can be alternatively spliced to create several different transcripts (GTEX, ENSG00000135486.17; NCBI, Gene ID 3178), including two that encode for the isoforms hnRNP A1 (NP_002127.1, CCDS: 41793.1, UniProt: P09651-2) and hnRNP A1B (NP_112420.1, CCDS: 44909.1, UniProt: P09651-1). The coding sequence of these two isoforms differs only by the inclusion of an alternatively spliced exon, referred to as exon 7B, which elongates the GRD by 52 amino acids. This splicing event can be modulated by hnRNP A1 itself and we have also previously shown that it is repressed by TDP-43 in cellular models (Chabot et al., 1997; Deshaies et al., 2018). hnRNP A1 is central to RNA metabolism including transcription, splicing, nuclear export, translation, and turnover (Jean-Philippe et al., 2013). However, very little is known about its alternative isoform hnRNP A1B.

The tight regulation of RBP expression is crucial for spatiotemporal regulation of RNA and is indelibly linked to development, differentiation and aging (Kamma et al., 1995; Bronstein et al., 2003; Blanchette et al., 2006; Huang et al., 2010; Sephton et al., 2012; Geuens et al., 2016; Kemmerer et al., 2018). Importantly, the mislocalization of RBPs, such as TDP-43, FUS and hnRNP A1, is a histopathological feature of several neurodegenerative diseases, including amyotrophic lateral sclerosis (ALS) and frontotemporal dementia (FTD) (Deshaies et al., 2018; Aksoy et al., 2020; de Boer et al., 2020). However, while the spatial and temporal regulation of TDP-43 and FUS have been reported (Sephton et al., 2010; Huang et al., 2013), hnRNP A1 and its isoform hnRNP A1B have not. Determining the spatial and temporal expression patterns of hnRNP A1B and hnRNP A1 in tissues will facilitate a better understanding of the function of these two isoforms and enable a better consideration of their relative contributions to disease. Here, we demonstrate that hnRNP A1B and hnRNP A1 are differentially expressed and localized in mouse tissues during development and throughout aging.

## Materials and Methods

### Animals

C57BL6/N mice of both sexes were used in this study at various ages. For each time point, three mice were collected for protein and RNA extraction and two mice were perfused for imaging. For protein and RNA extraction, tissues were dissected, snap frozen in liquid nitrogen and stored at −80°C until use. For immunohistochemistry, mice were transcardially perfused with 4% paraformaldehyde (PFA) and tissues were dissected, fixed, cryopreserved in OCT (Sakura, 4583) and stored at −80°C until use. Spinal cords (30 μm transversal, floating) and sciatic nerves (10 μm longitudinal, mounted on charged microscope slides) were sectioned via cryostat.

### Cell Culture

Primary cortical neurons were prepared from E18.5 C57BL/6N mouse embryos, exactly as previously published (Khalfallah et al., 2018). CB3 cells are mouse erythroleukemia cells where the endogenous *HNRNPA1* allele is inactivated due to a retroviral insertion. These cells were stably transfected to uniquely express mouse hnRNP A1 or hnRNP A1B cDNA, as previously described (Yang et al., 1994). CB3 cells were cultured in Minimum Essential Media (MEM) Alpha Modification (Fisher Scientific) supplemented with 10% FBS (Life Technologies), 1% Penicillin-Streptomycin (Wisent) and 50 mg/ml geneticin (Life Technologies) to maintain stable expression. HeLa and SH-SY5Y cells were cultured in Dulbecco’s high glucose modified Eagle medium (DMEM, Fisher Scientific) supplemented with 10% FBS and 1% L-glutamine (Sigma). For differentiation, SH-SY5Y were cultured in Dulbecco’s Modified Eagle’s Medium F-12 (DMEM F-12, Fisher Scientific) supplemented with 10% FBS, 1% L-glutamine (Sigma) and 1% Penicillin-Streptomycin (Wisent). They were differentiated with 10 μm retinoic acid (Tocris) and 50 ng/ml of Brain Derived Neurotrophic Factor (BDNF, Sigma) as published (Encinas et al., 2000).

### Characterization of hnRNP A1B Antibody

Polyclonal antibodies were commercially generated by Medimabs (Montreal, QC) in rabbits immunized with a synthetic peptide (C-YGGSGSYDSYNNGG), corresponding to amino acids 281-294 (encoded by exon 7B) of hnRNP A1B (NP_112420.1) (Supplementary Figure 1A). Sera were affinity purified using the same immunization peptide. To demonstrate specificity, affinity-purified antibody was pre-adsorbed with GST-tagged recombinant mouse hnRNP A1 or hnRNP A1B protein (generously provided by Dr. Benoit Chabot, U. Sherbrooke, QC) at a 1:10 ratio in PBS-T. This was incubated O/N at 4°C with rotation, then cleared with 4B glutathione sepharose beads (GE HealthCare).

### Immunohistochemistry

Antigen retrieval was performed on sections via heating to 90°C for 20 min in citrate buffer pH 6.0. Sections were then blocked with 5% donkey serum (Jackson ImmunoResearch) and then sequentially labeled with the indicated primary antibodies: anti-hnRNP A1B (custom, 1:100), anti-hnRNP A1 (4B10, Santa Cruz, 1:100) and donkey anti-mouse/rabbit secondary antibody conjugated with horseradish peroxidase (HRP, Jackson ImmunoResearch, 1:500). The signal was visualized with the DAB substrate-chromogen kit (Jackson ImmunoResearch) and slides were imaged with a bright field microscope (Leica DM4000B).

### Immunofluorescence

Tissue sections were blocked in 3% bovine serum albumin (BSA) in PBS and permeabilized with 0.5% Tween-20 in PBS, before incubation with primary antibody overnight at 4°C. Cells grown on coverslips were fixed with 4% PFA/PBS, permeabilized with 0.2% Triton X-100/PBS and blocked with 1%BSA/PBS before incubation with indicated primary antibody (anti-hnRNP A1B, anti-ChAT (Millipore,1:75), anti-hnRNP A1 (4B10, Santa Cruz, 1:50), anti-NF-H (Covance,1:1000), anti-myc (Sigma,1:1000), anti-GFAP (DakoCytomation,1:1000), anti-Iba1 (Novus Biologicals,1:200). Sections or coverslips were then incubated with the appropriate fluorophore conjugated secondary antibodies for 1h at RT, and then mounted using Prolong antifade reagent (ThermoFisher). Longitudinal nerve sections were counterstain with fluoromyelin (ThermoFisher, 1:300). Images were acquired using a Leica TCS SP5 confocal microscope equipped with 20× and 63× oil objectives and Leica Application Suite imaging software.

Immunofluorescent labeling at the NMJ was performed according to the method previously described (Arbour et al., 2017). Briefly, mice were sacrificed using isoflurane and *Soleus* muscles were dissected in oxygenated Rees’ Ringer’s solution and pinned in a Sylguard coated petri dish (Dow Corning). Muscles were then fixed for 10 min in 4% paraformaldehyde (PFA, Mecalab) diluted in PBS buffer at RT and permeabilized in 100% cold-methanol at −20°C for 6 min. Non-specific labeling was blocked by incubating muscles with 10% normal donkey serum in PBS containing 0.01% Triton X-100 for 20 min at RT. Muscles were then incubated overnight at 4°C with anti-hnRNP A1B, then 2 h at RT with anti-NF-M (Rockland Immunochemicals Inc, 1:2000) and anti-SV2 (Developmental Studies Hybridoma Bank, 1:2000). After washing, muscles were incubated with the appropriate secondary antibodies for 1 h, all together. Postsynaptic nicotinic acetylcholine receptors (nAChRs) were labeled with anti-bungarotoxin (Invitrogen, 1:500) for 45 min. Muscles were then mounted in Prolong Gold antifade reagent (Invitrogen). All labels were observed simultaneously using the spectral detection feature of an Olympus FV1000.

### Lysate Preparation

For P28 to 18M mice, cervical and lumbar spinal cords were microdissected on ice and lysed to extract protein and RNA using the Norgen extraction kit (Norgen Biotek, 47700), according to the manufacturer’s protocol. For younger time points, the whole spinal cord was lysed using the same kit. For P7 to 18M, frontal cortex and cerebellum were microdissected on ice while whole brain was used for the younger time points. These samples were lysed with the Norgen extraction kit to extract RNA. For protein extraction, samples were lysed in 50 mM Tris pH 7.5, 1 mM EDTA, 150 mM NaCl, 1% SDS and 1% NP-40 with protease inhibitors. Cells were lysed in RIPA buffer (50 mM Tris pH 7.4, 150 mM NaCl, 1% Triton X-100, 0.1% SDS, 1% sodium deoxycholate) with protease inhibitors. For the nucleocytoplasmic fractionation differentiated SH-SY5Y cells were collected and lysed to obtain cytoplasmic and nuclear fractions using a published protocol (Brugiolo et al., 2017).

### Immunoblot

After protein quantification by BCA (Pierce), equal amounts of 15 μg of lysates were subjected to 12.5% SDS-PAGE separation. Proteins were transferred to nitrocellulose membranes which were then blocked with 5% powdered milk/PBS-T before incubation with primary antibodies: anti-hnRNP A1B (custom; 1:2000), anti-actin (MP Biomedical, 1:10 000), anti-hnRNP A1 (4B10, Santa Cruz, 1:1 000), anti-TDP-43 (Proteintech, 1:10 000), anti-tubulin (Abcam, 1:1000). Membranes were then incubated with appropriate HRP-conjugated secondary antibody (Jackson ImmunoResearch, 1:5000) and signal was revealed by ECL (ThermoFisher Scientific). Acquisition was done on CL-Xposure radiography films (ThermoFisher Scientific), BIO-RAD ChemiDOC MP imaging system, or LiCor. Mean intensity was measured using Photoshop (Adobe) and normalized to a loading control.

### qRT-PCR

Equal amounts of RNA were reverse transcribed using the QuantiTect Reverse Transcription kit (Qiagen). The QuantStudio 7 Flex Real-Time PCR System (Life Technologies) was used for qPCR. PrimeTime Standard qPCR assays (IDT) were: *Actb* Mm.PT.39a.22214843.g (sequence); *RPLP0* Mm.PT.58.43894205 (sequence). To detect hnRNP A1 encoding transcripts, the following primer sets were used: forward: 5′-AGGTTCCACAACTCTTCCATC-3′, reverse: 5′-TGAGAGAT CCAAACACCAAGAG-3′; Probe 5′-TGGATGCTGCCATGA-3′. To detect hnRNP A1B encoding transcripts, the following primers were used: forward: 5′-CGCTCCTCCGTTGTTATAG-3′, reverse: 5′-AAGTGGTGGACAGGGTTATG-3′; probe: 5′ AGTGGCAGCTATG-3′. Data were analyzed using the 2^–ΔΔ^Ct method. The genes of interest were normalized using the geometric mean of two housekeeping genes (*Actb* and *RPLP0*).

### Size Exclusion Chromatography

Size exclusion chromatography (SEC) was performed as previously described (Sévigny et al., 2020). Mouse brains of P18 mice were processed using a Dounce homogenizer in homogenization buffer [1X Hank’s balanced salt solution (Gibco), 10 mM HEPES pH 7.3 (Gibco), 0.1 mg/ml cyclohexamide (Sigma)]. NP-40 was added to the homogenates to a concentration of 1% and incubated on ice for 10 min. Homogenates were centrifuged at 2,000 × *g* for 10 min at 4°C. The S1 was precleared by centrifugation at 20,000 × g for 10 min at 4°C. The S2 was filtered through a 0.45 μm filter and 25 mg of protein was loaded onto a Superdex 200 10/300 GL column. Samples were eluted using 20 mM Tris-HCl pH 7.4, 5 mM MgCl_2_ and 1 mM KCl buffer at a flow rate of 0.5 ml/min. A total of 48 fractions were collected at 0.5 ml/fraction and proteins from fractions 3-26 were processed for western blotting. Molecular mass calibration was carried out using the Gel Filtration Molecular Weight Markers Kit (Sigma-Aldrich).

### Analysis and Statistics

For immunohistochemistry images, the intensity of hnRNP A1 and hnRNP A1B was measured in arbitrary units across a segment with FIJI. Data are plotted to display intensity/distance and a gray box was drawn to highlight the nuclear region based on the Hoechst signal. All statistical analyses were performed using GraphPad Prism 9.0.2 and *p* < 0.05 was considered as significant. Values are reported as the mean with error bars representing the standard error (SEM). Immunoblot and qRT-PCR data were compared using a two-way ANOVA followed by a Tukey *post hoc* where time was selected as a variable. Simple comparisons were analyzed using a Student *t*-test.

## Results

### Characterization of an hnRNP A1B Specific Antibody

To analyze the spatial-temporal expression of hnRNP A1B specifically, an antibody that uniquely recognizes this alternative isoform is required. The most commonly used and commercially available antibodies for hnRNP A1 variably detect hnRNP A1B. Specifically, monoclonal antibody clone 4B10 preferentially recognizes hnRNP A1 with little to no recognition of hnRNP A1B (hereafter labeled as α-hnRNP A1) while monoclonal antibody clone 9H10 recognizes both isoforms (hereafter labeled as α-hnRNP A1/A1B). These differences in antibody reactivity can be partially explained by the targeted epitope; 4B10 targets the junction of exon 7 and 8 in hnRNP A1 (positions 251-262) while 9H10 targets a region in the shared exon 8 (position 287/339-302/354 in hnRNP A1/A1B) (Supplementary Figure 1A; Libner et al., 2020). There is no commercial antibody that specifically recognizes hnRNP A1B. To overcome this limitation, we generated a polyclonal hnRNP A1B-specific antibody which was raised against a peptide that is encoded uniquely by exon 7B (position 281-294) (α-hnRNP A1B) (Supplementary Figure 1A). To validate the selectivity of α-hnRNP A1B, we took advantage of CB3 cells that have inactivated *HNRNPA1* alleles (due to a retroviral insertion) and where the coding sequence of hnRNP A1 or hnRNP A1B has been stably reintroduced (Yang et al., 1994). We probed lysates from the different CB3 cell lines, as well as HeLa cells which express both isoforms simultaneously, with α-hnRNP A1B. α-hnRNP A1B recognized only the hnRNP A1B isoform at 38 kDa, and as previously reported, α-hnRNP A1 4B10 detected only the hnRNP A1 isoform at 34 kDa (Supplementary Figure 1B). We further validated that α-hnRNP A1B could preferentially recognize hnRNP A1B in its native state using immunoprecipitation. (Note, a minor amount of hnRNP A1 was immunoprecipitated but was only detectable following a long exposure). This revealed that our hnRNP A1B antibody preferentially recognizes hnRNP A1B in CB3-A1B cells and also in HeLa cells which express a mixture of both isoforms (Supplementary Figure 1C). The recognition of native hnRNP A1B is further validated by immunofluorescence where α-hnRNP A1B recognized endogenous hnRNP A1B, but its signal increased only when cells were transiently transfected to overexpress hnRNP A1B over hnRNP A1 (Supplementary Figure 1D). Finally, pre-adsorption of our custom antibody with recombinant hnRNP A1B protein demonstrated a loss of signal by both western blot (Supplementary Figure 1E) and immunohistochemistry (Supplementary Figure 1F). Taken together, these results demonstrate the specificity of our custom antibody for the hnRNP A1B isoform.

### hnRNP A1B Is Predominantly Expressed in the Central Nervous System as an SDS-Resistant Dimer

**FIGURE 1:**
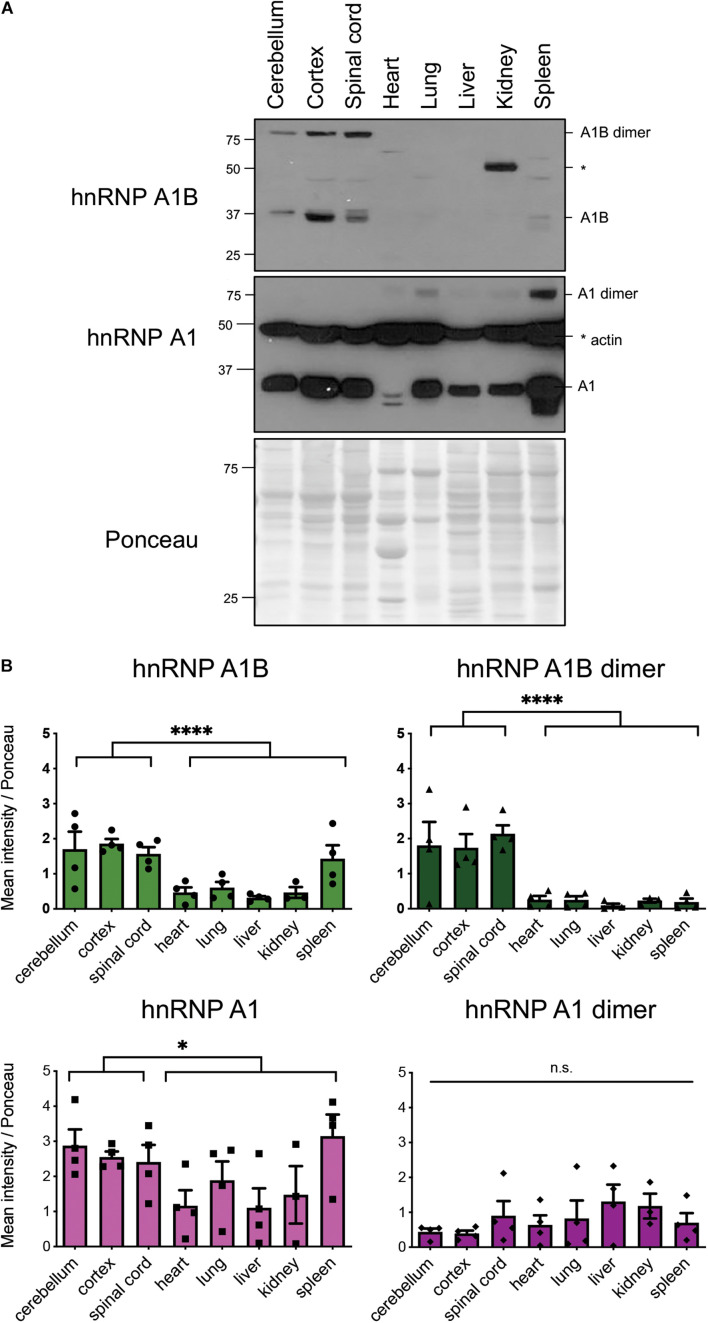
Expression of hnRNP A1B mouse tissue. **(A)** Representative immunoblot of tissue lysates of 2 month-old mice. **(B)** Quantification of mean intensity normalized to Ponceau S, data are expressed as the mean ± SEM, *n* = 4; Student *t*-test for CNS vs non-CNS tissues; *p < 0.05, ****p < 0.001.

Determining the tissue expression of different protein isoforms is essential to inform on differences in regulation and function. To determine if hnRNP A1B has an expression pattern that mirrors hnRNP A1, we examined a number of tissues collected from 2 month-old mice via immunoblotting with either α-hnRNP A1 or our newly characterized α-hnRNP A1B. Consistent with previous work (Hanamura et al., 1998; Liu et al., 2016; Deshaies et al., 2018), hnRNP A1 was highly expressed in the cortex, cerebellum, spinal cord, lung and spleen, and was expressed in other tissues to a lesser amount (Figures 1A,B). Evaluation of the same tissues using our hnRNP A1B-specific antibody revealed a similar pattern in the CNS for a 38 kDa band, consistent with the expected molecular weight for monomeric hnRNP A1B, albeit with less intense reactivity. Importantly, compared to hnRNP A1, hnRNP A1B was not detect in peripheral tissues (Figures 1A,B). In blots probed with α-hnRNP A1, we also observed a slower-migrating band in the peripheral tissues, whose size would be consistent with an hnRNP A1 dimer as proposed by *in vitro* work (Ding et al., 1999; Kooshapur et al., 2018), and which was under-represented in the CNS samples (Figures 1A,B). Interestingly, when blotting with α-hnRNP A1B, a similar slowly-migrating band was robustly detected in CNS tissues compared to the other tissues. We noted that this larger molecular weight form of hnRNP A1B migrated to a position consistent with an SDS-resistant dimer at 76 kDa. Interestingly, we did not observe a band at 72 kDa that would correspond to a hnRNP A1B-hnRNP A1 heterodimer (Figures 1A,B).

It has been previously reported that hnRNP A1 dimerizes via its UP1 domain to bind RNA in an antiparallel orientation (Ding et al., 1999; Kooshapur et al., 2018). Since this UP1 domain is conserved between hnRNP A1 and hnRNP A1B, we hypothesize that hnRNP A1B can also dimerize. Efforts to definitively identify this slower migrating band as hnRNP A1B using mass spectrometry were unsuccessful due to inefficient immunoprecipitation of the putative dimeric form (data not shown). So to investigate whether the slower migrating band detected by our newly generated antibody represents an SDS-resistant dimeric form of hnRNP A1B, we performed size exclusion chromatography of mouse brain lysates. This experiment revealed α-hnRNP A1B reactive bands in fractions corresponding to a multimeric pattern. Monomeric hnRNP A1 was identified at 34 kDa in fractions 21 to 24 and monomeric hnRNP A1B at 38 kDa in fractions 21 to 23. A multimeric pattern was observed for hnRNP A1B with a band at 76 kDa in fractions 15 to 18 (Supplementary Figure 2A). In contrast, even though hnRNP A1 has been reported to dimerize *in vitro*, an hnRNP A1 dimer was not observed here, consistent with Figure 1B where the hnRNP A1 dimer was only observed in non-neuronal tissues. We determined that the dimer is of high stability as reducing agents (TCEP, DTT, BME) and denaturants (heat, SDS, urea) were all unable to dissociate the dimer (data not shown). Thus, the collective data are consistent with the idea that hnRNP A1B forms a CNS-specific SDS-resistant dimer.

### Temporal Expression of hnRNP A1B and hnRNP A1 in Central Nervous System Lysates

**FIGURE 2:**
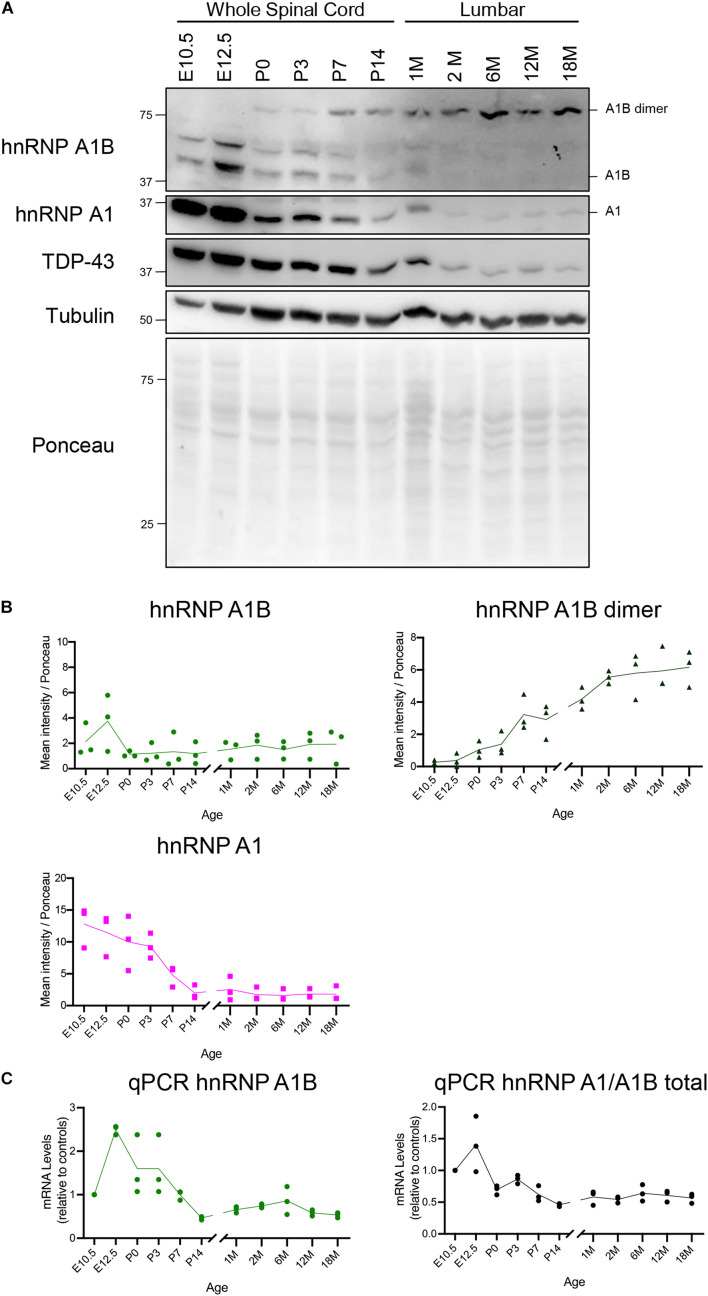
Expression of hnRNP A1B in the spinal cord throughout development and aging. **(A)** Immunoblot of mouse spinal cord at different ages, when possible the lumbar region was micro-dissected before lysis, blots are representative of *n* = 3. **(B)** Quantification of the mean intensity of hnRNP A1B (top left), dimeric hnRNP A1B (top right) and hnRNPA1 (bottom left) normalized to Ponceau, *n* = 3, two-way ANOVA (time variation: hnRNP A1B *p* = 0.1616, hnRNP A1B dimer *p* < 0.0001, hnRNP A1B dimer *p* < 0.0001). **(C)** qPCR of hnRNP A1B and hnRNP A1/A1B total mRNA levels expressed relative to the average of housekeeping control genes, *n* = 3, 2-way ANOVA (time variation: hnRNP A1B *p* = 0.0039).

Other hnRNPs have been shown to be temporally regulated during development and aging (Huang et al., 2010; Sephton et al., 2012; Ramos et al., 2019). Since hnRNP A1 and hnRNP A1B are predominantly expressed in the CNS, we examined their expression in mouse spinal cord and brain at ages ranging from embryonic day 10.5 to 18 months via immunoblotting. As previously reported, hnRNP A1 is highly expressed in the spinal cord of young mice and global levels gradually decrease in early juvenile ages, with little detectable protein in adult lumbar and cervical spinal cord lysates (2M-18M) (Figures 2A,B and Supplementary Figures 3A,B; Bronstein et al., 2003). Similarly, hnRNP A1B is readily detected in embryonic-aged samples, but its monomeric form decreases at P0. However, at this same age we detected a steady increase in the hnRNP A1B dimeric form such that there appears to be a complete shift from monomeric to dimeric hnRNP A1B in the spinal cord (time factor *p* < 0.0001). This temporal decline in expression after the developmental stage is mirrored by the mRNA levels of total hnRNP A1/A1B transcripts and unique hnRNP A1B transcripts, which are higher in the younger ages followed by a decline in the young adult animals (Figure 2C). This result also indicates that in aged samples, there is a steady and low abundance of transcripts, consistent with the residual protein expression observed in adulthood and throughout aging. A similar pattern was observed in cervical spinal cord (Supplementary Figure 3C) and similar tendencies were observed in the brain frontal cortex and cerebellum (Supplementary Figure 4). Lastly, as reported by others (Huang et al., 2010; Sephton et al., 2010), TDP-43 protein levels in the spinal cord declined similarly with age (time factor *p* = 0.0005) (Figure 2A).

### Spatial Expression of hnRNP A1B in Spinal Motor Neurons With Aging

**FIGURE 3:**
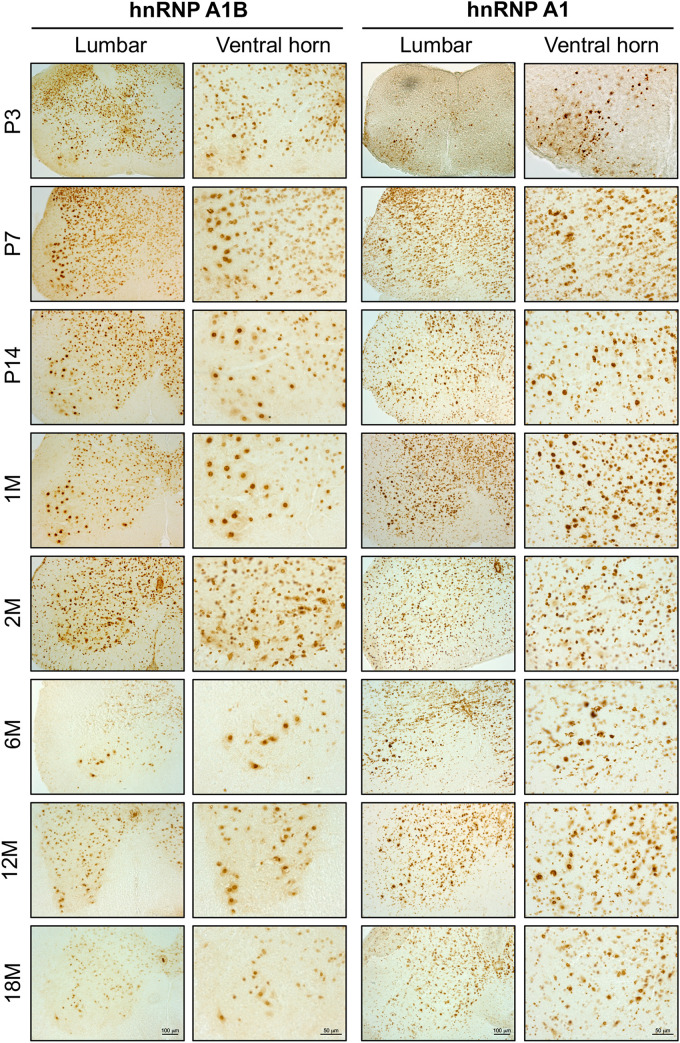
hnRNP A1B distribution in the spinal cord with age. Immunohistochemistry of mouse lumbar spinal cord at different time points (from post-natal day 3 to 18 months) labeled with α-hnRNP A1B and α-hnRNP A1 antibodies. Images are representative of 3 different sections from two animals, Lumbar = 10×, Ventral horn = 20×, scale bar corresponds to 100 μm for lumbar sections and 50 μm for ventral horn sections.

**FIGURE 4:**
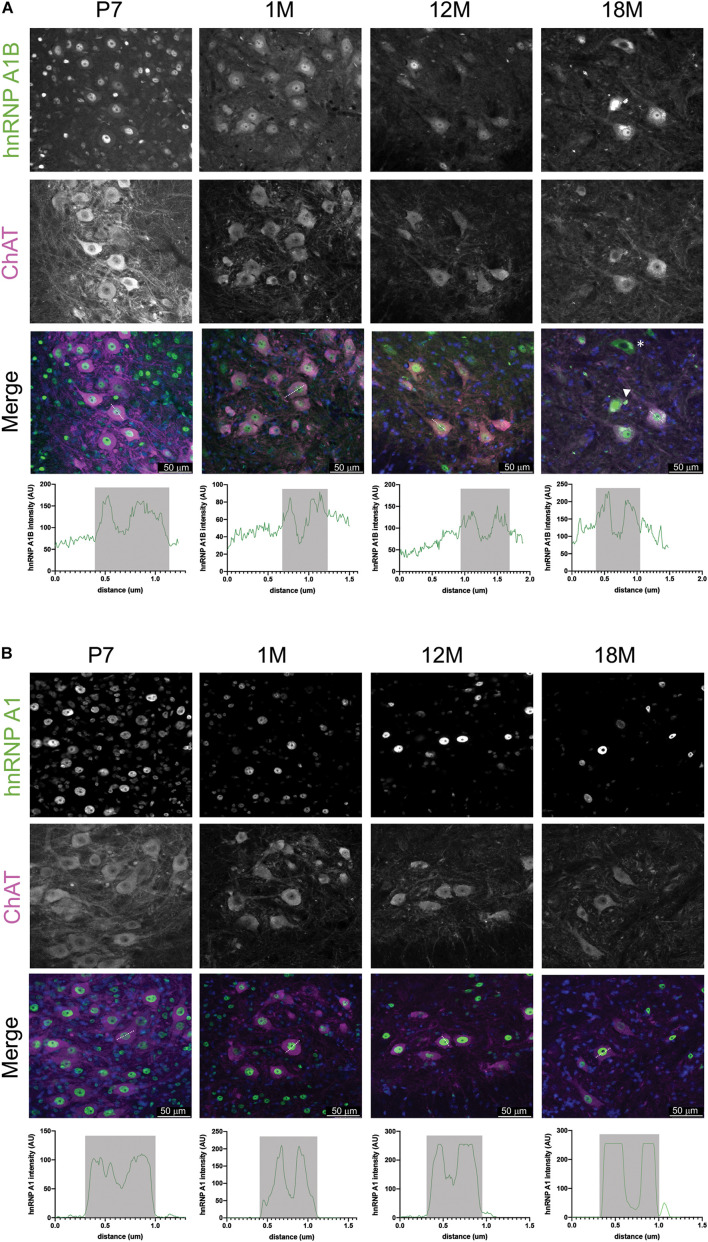
hnRNP A1B cellular distribution in motor neurons. **(A,B)** Immunofluorescence of mouse lumbar spinal cord with α-hnRNP A1B **(A)** and α-hnRNP A1 **(B)** (in green) co-labeled with ChAT (magenta) to identify motor neurons from ventral horn of spinal cord with a magnification of 20×. Representative images of staining from 3 sections from 2 animals. Scale bar = 50 μm. Arrowhead in merged images indicates cells with aggregation, asterisk represent cells with nuclear depletion, crossing lines indicates representative cells use for Z-plot distribution, Z-plot displaying α-hnRNP A1B and α-hnRNP A1 intensity (AU = arbitrary units) across a representative motor neuron, gray box indicates nuclear area.

Given that we have previously reported that hnRNP A1B is detectable in healthy control and ALS patient spinal motor neurons (Deshaies et al., 2018), we examined the spatial expression of hnRNP A1B (and hnRNP A1) in mouse lumbar spinal cords during normal aging using immunohistochemistry. As evidenced in the low power magnifications and consistent with our immunoblotting data, overall expression levels of hnRNP A1 and hnRNP A1B are highest in the spinal cords of young animals but decrease with age (Figure 3). In addition, hnRNP A1 and hnRNP A1B expressing cells were broadly distributed throughout the lumbar spinal cord in young animals, including the dorsal part of the lumbar spinal cord (Supplementary Figure 5). However, while hnRNP A1 expression remains broadly expressed across the spinal cord throughout adulthood, hnRNP A1B expression appeared to become progressively restricted to a subset of large cells in the ventral horn which we predicted to be motor neurons based on their morphology and size. To confirm this, we performed immunofluorescence for hnRNP A1B and the motor neuron marker ChAT (Figure 4A). hnRNP A1B colocalized with ChAT-positive cells at all time points examined and we noted that non-neuronal expression of hnRNP A1B decreased starting at 1 month. The same pattern was observed for hnRNP A1 (Figure 4B). hnRNP A1 and hnRNP A1B were not enriched in other CNS cell types, such as astrocytes or microglia (Supplementary Figure 6).

Closer inspection of lumbar motor neurons revealed that hnRNP A1B localized to the nucleus and a portion of it was also found in the cytoplasm, while hnRNP A1 showed nearly exclusive nuclear labeling (as expected), at all ages examined. Those phenotypes were consistent between the different sections examined and also within the frontal cortex (data not shown). Finally, at advanced age (18M), cytoplasmic hnRNP A1B aggregates were detected in some motor neurons (Figure 4A, example indicated by an arrow), while other motor neurons showed hnRNP A1B cytoplasmic labeling and nuclear depletion (Figure 4A, example indicated by an asterisk). Thus, both isoforms are robustly expressed in motor neurons throughout aging but demonstrate differential subcellular localization.

### Cytoplasmic hnRNP A1B Is Granular

**FIGURE 5:**
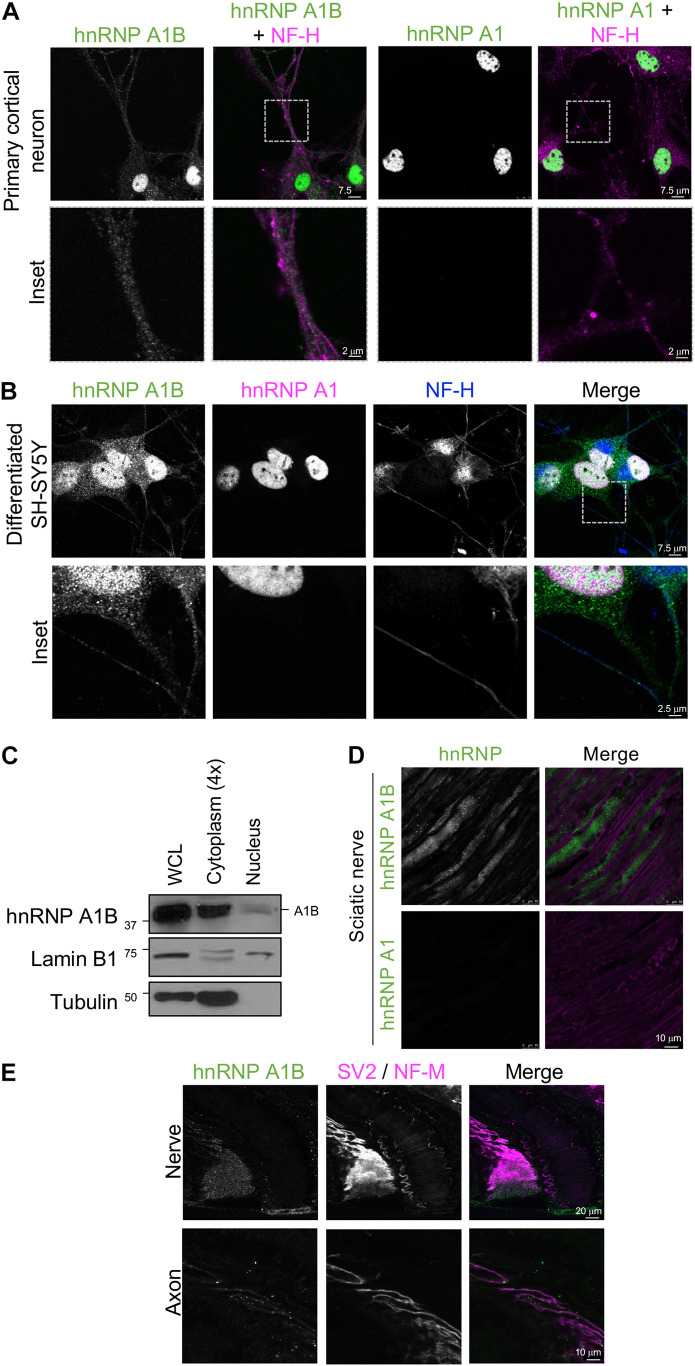
Localization of hnRNP A1B in the processes of neurons. **(A)**. Primary cortical neuron stained with α-hnRNP A1B and α-hnRNP A1 (green) co-labeled with α-NF-H (magenta), after 10 days of culture, imaged at 63×, scale bar = 7.5 μm. inset highlighting the axon is shown at the bottom, representative image of *n* = 3. **(B)**. Differentiated human SH-SY5Y cells were labeled with α-hnRNP A1B (green), α-hnRNP A1 (magenta), and α-NF-H (blue), representative image of *n* = 3, scale bars = 7.5 and 2.5 μm. **(C)**. Representative immunoblot of SH-SY5Y whole cell lysate (WCL), cytoplasmic, and nuclear fractions. Note, the cytoplasmic fraction was loaded 4× the nuclear fraction to ease visualization of the cytoplasmic hnRNP A1B pool. **(D)**. Immunofluorescence of longitudinal section of the sciatic nerve of 12M old mice with α-hnRNP A1B and α-hnRNP A1 (green) co-stained with fluoromyelin (magenta), scale bar = 10 μm, representative image of 4 sections from 2 different animals. **(E)**. Immunofluorescence of the soleus muscle innervation with α-hnRNP A1B (green) co-labeled with α-SV2 and α-NF-M (magenta) as pre-synaptic markers, representative image of *n* = 4, scale bar = 20 and 10 μm.

To have a more detailed examination of the localization of hnRNP A1B in neuronal soma or processes, we performed immunofluorescence of primary cortical neurons labeled with NF-H. Consistent with our observations in tissues, hnRNP A1B was detected in the nucleus and soma as well as in neuronal processes. In contrast, hnRNP A1 was nearly exclusive to the nucleus (Figure 5A). We also noted that hnRNP A1B expression in the neuronal processes was heterogenous, displaying a granular pattern consistent with RNPs (Figure 5A, *inset*). Similar results were observed in differentiated SH-SY5Y (human neuroblastoma) cells, supporting that cytoplasmic hnRNP A1B is conserved between species (Figures 5B,C). To further validate the presence of hnRNP A1B in neuronal extensions *in vivo*, we evaluated its localization in longitudinal sciatic nerve sections stained with fluoromyelin. Here, hnRNP A1B was clearly intra-axonal and detected in a granular pattern showing no colocalization with the myelin, while hnRNP A1 was completely absent (Figure 5D). Given the role of RBPs in the development and maintenance of the neuromuscular junction (NMJ) (Crawford Parks et al., 2017; Picchiarelli et al., 2019; Picchiarelli and Dupuis, 2020), we also examined hnRNP A1B expression along the length of a motor axon as it enters the soleus muscle. hnRNP A1B was detected in the nerve bundle and was observable as puncta all along the axon (Figure 5E). However, the signal gradually faded, and as the nerve became more ramified, hnRNP A1B was not detected at the pre-synaptic NMJ (Supplementary Figure 7). Collectively, these results suggest a localization of hnRNP A1B in neuronal processes that is not shared with hnRNP A1.

## Discussion

In this study, we have demonstrated that hnRNP A1B expression is largely restricted to the CNS compared to the commonly studied isoform hnRNP A1. This result supports the hypothesis put forward by others that isoform usage contributes to tissue-specific diversity (Xu et al., 2002; Weyn-Vanhentenryck et al., 2018). Studies of *HNRNPA1* alternative splicing in different vertebrate species reveals that hnRNP A1B is predominant in chicken and frog tissues. In contrast, hnRNP A1 arises in mouse and human, indicating that the exclusion of exon 7B is a mammalian-specific alternative splicing event and that hnRNP A1B is the ancestral RBP of the two (Gueroussov et al., 2017). Interestingly, neural alternative splicing events are enriched in genes associated with synaptic transmission, axon guidance, neural development and actin cytoskeleton reorganization and are highly conserved in vertebrate nervous systems, supporting their functional importance (Barbosa-Morais et al., 2012). In line with the observation that tissue-specific alternative splicing events often occur in IDRs (Barbosa-Morais et al., 2012; Gueroussov et al., 2017), our data demonstrate that hnRNP A1B is selectively expressed in the CNS, suggesting a crucial CNS-related function.

We detected hnRNP A1B in the adult mouse CNS as an SDS-resistant dimer. It has been previously reported that hnRNP A1B exhibits homotypic interactions, similar to hnRNP A1 (Gueroussov et al., 2017). To our knowledge, this is the first report of the hnRNP A1B dimer in the mouse CNS, thus we examined the literature for additional supporting evidence. In the giant axons of squid, an ortholog of hnRNP A1/A1B is reported as an SDS-resistant dimer (Lico et al., 2010, 2015; Lopes et al., 2019). Intriguingly, all of the known hnRNP A1/A1B domains are conserved in the squid orthologs (named hnRNP A/B prot.1; uniport ID: A0A0P0C6T8, and prot. 2; uniport ID: A0A0P0BZX3) with the squid proteins having an elongated IDR. Inspection of the sequences suggests that the squid proteins are more similar to human hnRNP A1B than to hnRNP A1 (Supplementary Figure 2B). Indeed, the conservation identity of squid prot. 1 and 2 increased when compared to exon 7B instead of the whole hnRNP A1 or A1B sequence, meaning that the GRD expansion is conserved. This also reinforces the concept that exon 7B skipping may have evolved as a mammalian-specific alternative splicing event.

Other RBPs have also been reported to dimerize and this dimerization is known to modulate their function. For example, another member of the hnRNP family, hnRNP C, can multimerize which improves its binding to RNA by a cooperative RRM dimer binding model (Cieniková et al., 2015). TDP-43 can also dimerize via its N-terminal region (RBD) (Vivoli-Vega et al., 2020). Interestingly, TDP-43 dimers are enriched in the cytoplasm and found in physiological conditions as well as in ALS patient brains (Shiina et al., 2010). TDP-43 dimerization serves to increase its DNA binding affinity and splicing activity (Chang et al., 2012; Jiang et al., 2017; Mompeán et al., 2017). Indeed, hnRNP A1 function is also dependent on its structure, as the position of its RRM domain affects its binding to target RNA (Beusch et al., 2017; Liu et al., 2017; Levengood and Tolbert, 2018). This could indicate that the hnRNP A1B dimer plays a role different from the monomeric form. These results highlight the importance to study tissue specific expression of RBPs.

We have characterized the longitudinal expression of hnRNP A1B in the CNS of mice and compared it to the more commonly studied isoform, hnRNP A1. Both hnRNP A1B and hnRNP A1 are highly expressed in the CNS during embryonic and postnatal development, but their global expression gradually decreases in adulthood reaching a consistent low level. This is similar to what has been reported for other RBPs, such as TDP-43 and FUS (Huang et al., 2010; Sephton et al., 2010). Moreover, we observed that hnRNP A1B expression becomes progressively restricted to motor neurons in the ventral spinal cord (Figure 3). Even if we consider that ChAT-positive cells decline with aging (Luine et al., 1986), the observed decrease in hnRNP A1B levels is not explained by neuronal loss as it is the non-neuronal signal that decreases over time (Stiles and Jernigan, 2010). These results demonstrate that there is an age-dependent inverse effect on RBP expression which is important to take into account when studying age-related diseases. Also, cell-type specification of hnRNP expression is not uncommon (Kamma et al., 1995; Liu et al., 2008), and thus, how this regulation is mediated should be further explored.

Our work also demonstrates that hnRNP A1 and hnRNP A1B display different subcellular distributions. As reported in the literature, hnRNP A1 constantly shuttles between the nucleus and the cytoplasm, but at steady-state it is observed to be primarily nuclear (Figure 5A). From previous literature, it is known that hnRNP A1 exits the nucleus to export newly synthesized mRNA to the cytoplasm (Vautier et al., 2001) and it reenters the nucleus via transportin-1 (TNPO-1) binding to its M9 motif (Siomi and Dreyfuss, 1995). However, upon specific stimuli, hnRNP A1 can be found in the cytoplasm due to phosphorylation of the M9 sequence which inhibits TNPO-1 binding (Lichtenstein et al., 2001; Allemand et al., 2005; Guil et al., 2006). Surprisingly, even though hnRNP A1 and hnRNP A1B share the same M9 sequence, hnRNP A1B has a basal cytoplasmic distribution as well as a nuclear pool. The mechanism that allows a proportion of hnRNP A1B to remain cytoplasmic is unknown. Several questions/hypotheses are possible, including: does hnRNP A1B bind TNPO-1 with the same affinity as hnRNP A1 or does structural folding prevent binding? Is the M9 region of a pool of hnRNP A1B constitutively phosphorylated so as to prevent its interaction with TNPO-1? Do other post-translational modifications participate in cytoplasmic retention of hnRNP A1B? Or is there competitive binding between TNPO-1 and hnRNP A1B cytoplasmic interacting protein partners? Further work is needed to address these questions.

While the level of the cytoplasmic pool of hnRNP A1B appears to be stable in adulthood, at advanced ages its distribution is more variable. Specifically, we observed neurons with exclusively cytoplasmic hnRNP A1B (i.e., hnRNP A1B nuclear depletion) as well as neurons showing nuclear hnRNP A1B and cytoplasmic hnRNP A1B aggregates (Figure 4A). These distribution changes observed with aging are not specific to hnRNP A1B. A similar age-related change in subcellular distribution is reported for TDP-43 (Termsarasab et al., 2020). This change in distribution could be explained by deficits in the nucleocytoplasmic transport machinery which is reported to decline during aging and in certain disease contexts (Mertens et al., 2015; Kim and Taylor, 2017; Eftekharzadeh et al., 2018). Indeed, the phenotypes we observed are reminiscent of neurodegenerative disorders that exhibit mislocalization and aggregations of RBPs and emphasize a similarity between aging and neurodegeneration. Moreover, our results are similar to what we have previously reported, that hnRNP A1B can be found in cytoplasmic aggregates in ALS patient motor neurons (Deshaies et al., 2018).

It has been hypothesized that exclusion of ancestral constitutive exons enriched in IDR residues has evolved in a mammalian tissue-specific manner in order to modulate protein interactions and localization, as well as to expand their splicing regulatory capacities ([Bibr B12][Bibr B81]; Gueroussov et al., 2017). For example, for hnRNP D, the skipping of exon 7 liberates hnRNP D from the nucleus to facilitate its function in other cellular compartments (White et al., 2013). Indeed, we speculate that the different subcellular localization of hnRNP A1B may be relevant to neuronal cytoplasmic functions and note that the hnRNP A1B puncta we observe in neuronal processes/axons is similar to the RNP particles observed in neurons (Knowles et al., 1996; Köhrmann et al., 1999; Tang et al., 2001; Huang et al., 2003; Zhang et al., 2006). Moreover, squid hnRNP A1B orthologous proteins are enriched in synaptosomes (Lopes et al., 2020). Taken together, we speculate that the cytoplasmic pool of hnRNP A1B in motor neurons is implicated in neuronal mRNA transport and/or translation (Kiebler and Bassell, 2006; Pushpalatha and Besse, 2019). Future studies are required to explore this novel function.

## Conclusion

This study provides the first detailed spatiotemporal characterization of two isoforms of hnRNP A1 throughout development and aging. The sustained presence of hnRNP A1B in the processes of neurons suggests that hnRNP A1B has a novel function that is not shared with hnRNP A1. It will inform studies of their misregulation in disease contexts. Finally, our results reinforce the idea that RBP isoform usage, expression and localization are continuously regulated and that a deeper understanding of RBP spatiotemporal regulation is critical to inform on physiology and pathology.

## Data Availability Statement

The raw data supporting the conclusions of this article will be made available by the authors, without undue reservation.

## Ethics Statement

The animal study was reviewed and approved by Institutional Committee for the Protection of Animals (CIPA) of the Centre de Recherche du Centre Hospitalier de l’Université de Montréal (CRCHUM).

## Author Contributions

MG, J-ED, and CVV contributed to the conception and design of the study. MG and SP collected the animals. MG, GL, and SP processed IHC samples for imaging. HS contributed to tissue lysis and performed qRT-PCR. DA and RR performed NMJ staining. YB and CS performed the SEC. MG collected and analyzed all other data. AD contributed to important discussions. MG and CVV wrote the manuscript. All authors read and approved the final manuscript.

## Conflict of Interest

The authors declare that the research was conducted in the absence of any commercial or financial relationships that could be construed as a potential conflict of interest.

## Publisher’s Note

All claims expressed in this article are solely those of the authors and do not necessarily represent those of their affiliated organizations, or those of the publisher, the editors and the reviewers. Any product that may be evaluated in this article, or claim that may be made by its manufacturer, is not guaranteed or endorsed by the publisher.

## Supplementary Material

Supplementary Figure 1 Validation of homemade hnRNP A1B antibodies. **(A)** Schematic of *HNRNPA1* (Gene ID: 3178) alternative splicing of exon 7B to form hnRNP A1B or hnRNP A1 isoforms. Protein domains are shown in the different isoforms; RRM1 and RMM2: RNA recognition motif 1 and 2, RGG: Arginine-glycine-glycine repeat domain, 7B; exon 7B, GRD: glycine-rich domain, M9: PY-NLS sequence responsible for nucleocytoplasmic shuttling. Epitope mapping of commercial hnRNP A1 antibodies (4B10 and 9h10) (Libner et al., 2020) and representation of hnRNP A1B unique peptide use to inoculate rabbit (253) to produce hnRNP A1B specific antibodies. **(B)** hnRNP A1B antibody specificity validation by immunoblot on CB3 cells that are endogenous knockout of *HNRNPA1* and where the coding sequence of hnRNP A1 or hnRNP A1B has been stably expressed (Yang et al., 1994) and on HeLa that expresses both isoforms at a basal state. **(C)** hnRNP A1B antibody specificity validation by immunoprecipitation, whole cell lysate of different cell lines were subjected to hnRNP A1B antibody coupled to Dynabeads protein G and immunoblotted with α-hnRNPA1/A1B (9H10). **(D)** hnRNP A1B antibody specificity validation by immunofluorescence in HeLa transfected with either myc-hnRNPA1 or myc-hnRNP A1B and labeled for α-hnRNP A1B (green), α-myc (magenta), α-hnRNP A1 4B10 (blue). **(E)** Immunoblot of CB3 WCL and **(F)** immunohistochemistry of lumbar spinal cord section of 12M old mice where homemade antibody has been pre-adsorbed with mouse GST-hnRNP GST-hnRNP A1B recombinant protein.

Supplementary Figure 2 hnRNP A1B can form a dimer. **(A)** Immunoblot of fractions from SEC of P18 brain, showing that hnRNP A1B can multimerize. **(B)** Sequence alignment representing squid hnRNPA/B protein (1, 2 respectively) (Lico et al., 2015) and human hnRNP A1/A1B, main protein domains are illustrated by colored boxes [RBD = RNA binding domain, RBD1 and RBD2 = RRM in human (orange), RGG (purple), 7B exon (green), M9 sequence (red)], symbols legend (**^∗^** = fully conserved residue,: = conservation between groups of strongly similar properties,. = conservation between groups of weakly similar properties).

Supplementary Figure 3 Expression of hnRNP A1B in the cervical spinal cord during development and aging. **(A)** Immunoblot mice cervical region of the spinal cord at different ages, blots are representative of *n* = 3. **(B)** Quantification of mean intensity normalized on Ponceau, *n* = 3. **(C)** qPCR of hnRNP A1B and hnRNP A1/A1B total mRNA levels expressed relative to the average of controls, *n* = 3.

Supplementary Figure 4 Expression of hnRNP A1B in the brain over development and age. **(A)** Immunoblot of mice brain at different ages, when possible, cerebellum and frontal cortex were micro-dissected before lysis, blots are representative of *n* = 3. **(B)** Quantification of mean intensity normalized on Ponceau, *n* = 3, 2-way ANOVA (time variation: hnRNP A1B *p* < 0.0001, hnRNP A1B dimer *p* < 0.0344, hnRNP A1 *p* = 0.0685). **(C)** qPCR of hnRNP A1B and hnRNP A1/A1B total mRNA, level relative to the average of controls, *n* = 3, 2-way ANOVA (time variation: hnRNP A1B *p* = 0.0044).

Supplementary Figure 5 hnRNP A1B labeling of dorsal horn neurons of the lumbar spinal cord. Immunofluorescence of lumbar spinal cord of mice with **(A)**. α-hnRNP A1B and **(B)**. α-hnRNP A1 antibodies (green), co-labeled with α-ChAT (magenta), dorsal 20×, scale bar = 100 μm.

Supplementary Figure 6 hnRNP A1B is not detected in astrocytes and microglia in the ventral spinal cord. **(A)** Immunofluorescence of lumbar spinal cord of 1M mice with α-hnRNP A1B (left) and α-hnRNP A1 (right) labeled in green and co-labeled with α-GFAP (magenta) or **(B)**. α-Iba1 (magenta), ventral 20X scale bar = 100 μm, scale bar = 50 μm for the magnified images.

Supplementary Figure 7 hnRNP A1B is not detected at the neuromuscular junction. Immunofluorescence of the NMJ in the soleus with α-hnRNP A1B (green) co-labeled with α-SV2 and α-NF-M (magenta) as pre-synaptic markers and α-bungarotoxin (blue), representative image of *n* = 4, scale bar = 20 and 10 μm.

## Abbreviations

A1: heterogenous ribonucleoprotein A1
A1B: heterogenous ribonucleoprotein A1B
CB3 A1/A1B: mouse erythroleukemia cell line CB3C7 stably expressing mouse hnRNP A1/A1B
CB3: mouse erythroleukemia cell line CB3C7
ChAT: choline acetyltransferase
CNS: central nervous system
DNA: deoxyribonucleic acid
FUS: fused in sarcoma
GFAP: glial fibrillary acidic protein
GRD: glycine-rich domain
GST-A1/A1B: recombinant mouse hnRNP A1/A1B protein tagged with glutathione S-transferase
hnRNP: heterogenous ribonucleoprotein
Iba1: ionized calcium-binding adapter molecule
IDR: intrinsically disordered region
NF-H: Neurofilament-heavy chain
NF-M: Neurofilament-medium chain
NLS: nuclear localization sequence
NMJ: neuromuscular junction
RBD: RNA binding domain
RBP: RNA binding protein
RGG: Arginine-glycine-glycine repeat domain
RNA: ribonucleic acid
RNP: ribonucleoprotein
RRM: RNA recognition motif
SEC: size exclusion chromatography
SV2: synaptic vesicular protein 2
TDP-43: transactive response DNA-binding protein 43
TNPO-1: transportin-1.
