# Supplementary material for: hnRNP A1B, a Splice Variant of HNRNPA1, Is Spatially and Temporally Regulated
Source: Front Neurosci. 2021 Sep 24;15:724307. doi: 10.3389/fnins.2021.724307 (PMC8498194; doi:10.3389/fnins.2021.724307)
Supplement: Supplementary Figure 1 — Validation of homemade hnRNP A1B antibodies. (A) Schematic of HNRNPA1 (Gene ID: 3178) alternative splicing of exon 7B to form hnRNP A1B or hnRNP A1 isoforms. Protein domains are shown in the different isoforms; RRM1 and RMM2: RNA recognition motif 1 and 2, RGG: Arginine-glycine-glycine repeat domain, 7B; exon 7B, GRD: glycine-rich domain, M9: PY-NLS sequence responsible for nucleocytoplasmic shuttling. Epitope mapping of commercial hnRNP A1 antibodies (4B10 and 9h10) (Libner et al., 2020) and representation of hnRNP A1B unique peptide use to inoculate rabbit (253) to produce hnRNP A1B specific antibodies. (B) hnRNP A1B antibody specificity validation by immunoblot on CB3 cells that are endogenous knockout of HNRNPA1 and where the coding sequence of hnRNP A1 or hnRNP A1B has been stably expressed (Yang et al., 1994) and on HeLa that expresses both isoforms at a basal state. (C) hnRNP A1B antibody specificity validation by immunoprecipitation, whole cell lysate of different cell lines were subjected to hnRNP A1B antibody coupled to Dynabeads protein G and immunoblotted with α-hnRNPA1/A1B (9H10). (D) hnRNP A1B antibody specificity validation by immunofluorescence in HeLa transfected with either myc-hnRNPA1 or myc-hnRNP A1B and labeled for α-hnRNP A1B (green), α-myc (magenta), α-hnRNP A1 4B10 (blue). (E) Immunoblot of CB3 WCL and (F) immunohistochemistry of lumbar spinal cord section of 12M old mice where homemade antibody has been pre-adsorbed with mouse GST-hnRNP GST-hnRNP A1B recombinant protein. [file Data_Sheet_1.PDF]

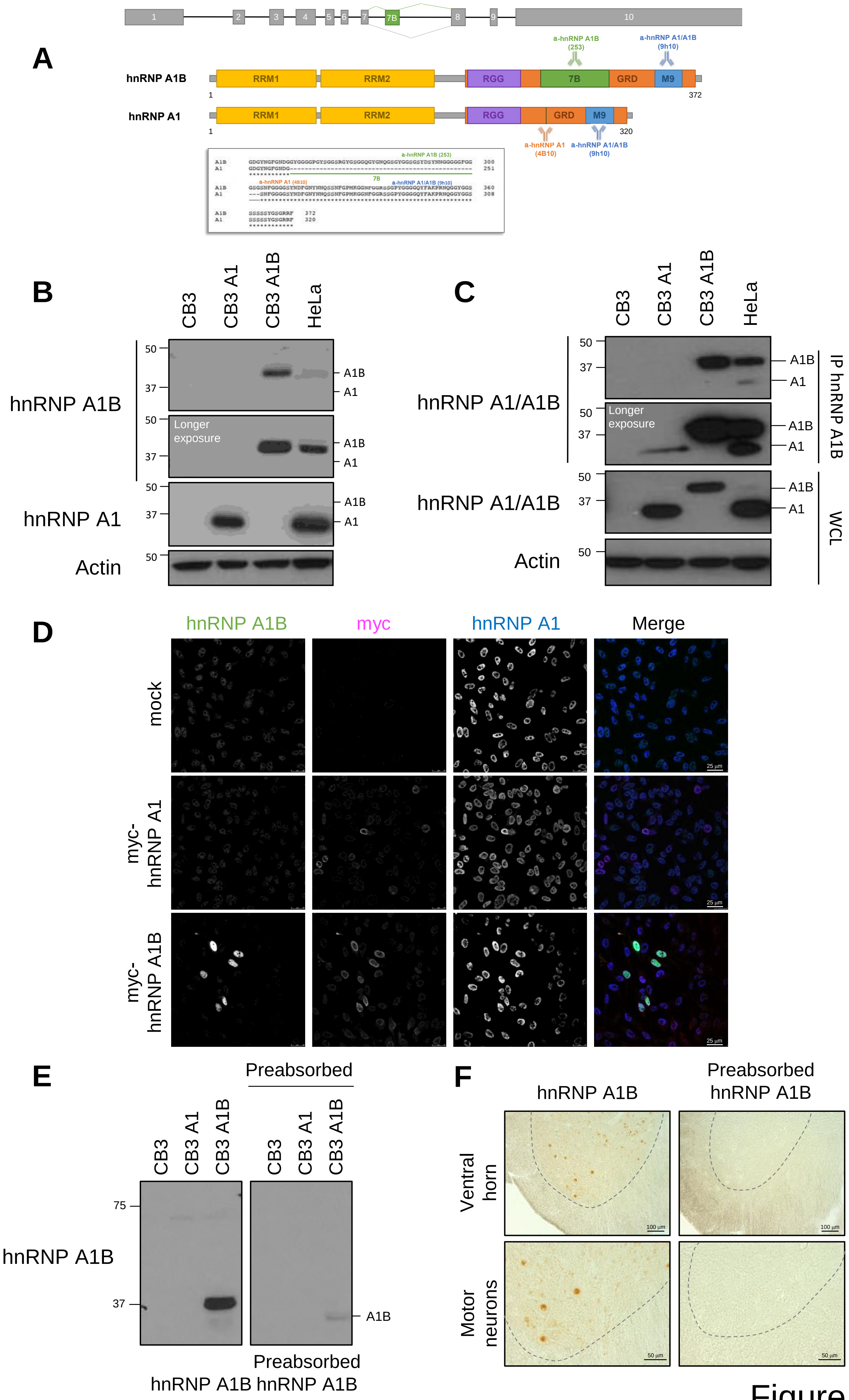
A
hnRNP A1B
hnRNP A1
RRM1
RRM2
RGG
7B
GRD
M9
a-hnRNP A1B (253)
a-hnRNP A1/A1B (9h10)
a-hnRNP A1 (4B10)
1
372
320
B
CB3
CB3 A1
CB3 A1B
HeLa
hnRNP A1B
Longer exposure
hnRNP A1
Actin
A1B
A1
C
hnRNP A1/A1B
IP hnRNP A1B
WCL
D
hnRNP A1B
myc
hnRNP A1
Merge
mock
myc-hnRNP A1
myc-hnRNP A1B
25 µm
E
Preabsorbed
75
37
Preabsorbed hnRNP A1B
hnRNP A1B
F
Ventral horn
Motor neurons
100 µm
50 µm

Figure S1

A

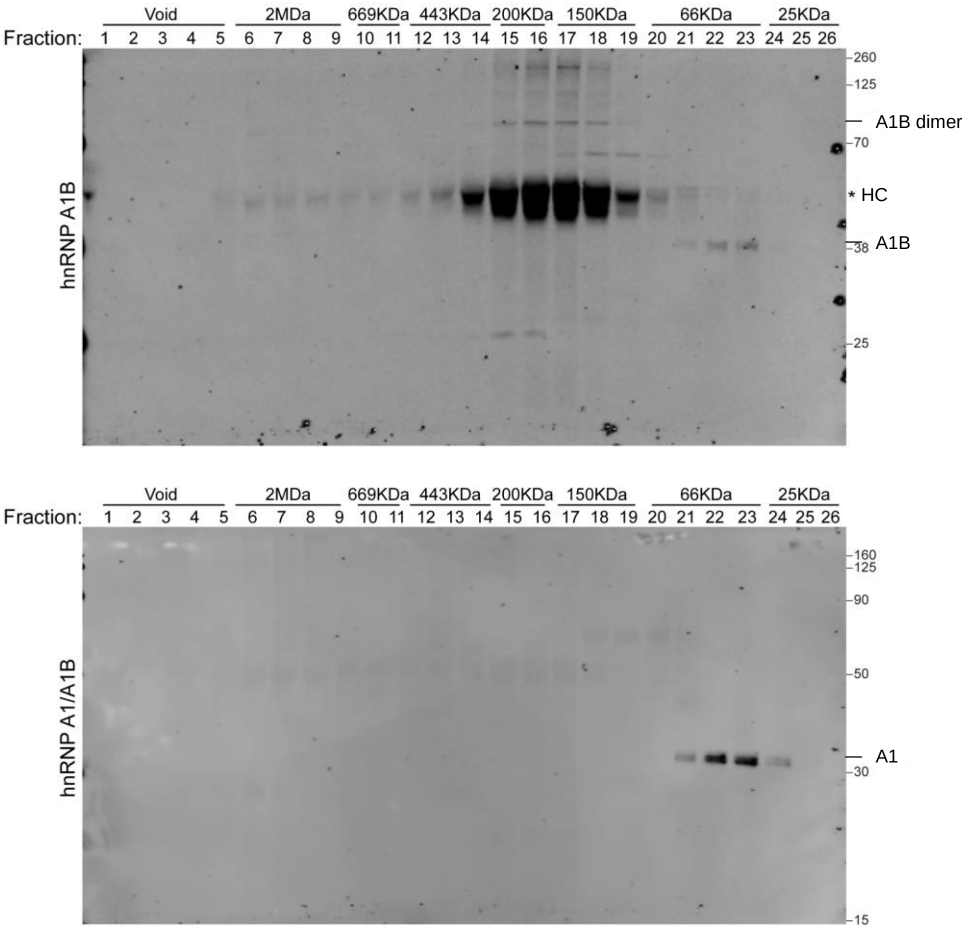

B

```
                                                      RBD2
human_HNRNPA1        -------MSKSESPKEPEQLRKLFIGGLSFETTDESLRSHFEQWGTLTDCVVMRDPNTKR      53
human_HNRNPA1B       -------MSKSESPKEPEQLRKLFIGGLSFETTDESLRSHFEQWGTLTDCVVMRDPNTKR      53
squid_prot1          MPESTGRYRDS--DSDGEKFCKFFIGGLNYTTNEEAMKEYFEPWGEVVDCVVMRDPNTKK      58
squid_prot2          MPERYNSYRDDNDDPQAEKFRKLFIGGLNYDTTEETIKQHFEQWGEIVDCVVMKNPATKK      60
                              ..     : *:: *:*****.: *.:*:::.:** ** :.*****::* **:
                       RBD1                                                  RBD2
human_HNRNPA1        SRGFGFVTYATVEEVDAAMNARPHKVDGRVVEPKRAVSREDSQRPGAHLTVKKIFVGGIK     113
human_HNRNPA1B       SRGFGFVTYATVEEVDAAMNARPHKVDGRVVEPKRAVSREDSQRPGAHLTVKKIFVGGIK     113
squid_prot1          SRGFGFITYKTEEQVDEAQRNRPHNIDNKEVETKRARPRNETD---SQATVKKLFVGGIK     115
squid_prot2          SRGFGFITYKAAEMLDDAQTNRPHKIDNRELDTKRAMPRNESDE--TQASVKKMFVGGLK     118
                     ******:** : * :* *   ***::*.: :: ***  *::::   :: :***:****:*
                                                         RBD1
human_HNRNPA1        EDTEEHHLRDYFEQYGKIEVIEIMTDRGSGKKRGFAFVTFDDHDSVDKIVIQKYHTVNGH     173
human_HNRNPA1B       EDTEEHHLRDYFEQYGKIEVIEIMTDRGSGKKRGFAFVTFDDHDSVDKIVIQKYHTVNGH     173
squid_prot1          EDTSEDEIREFFSTKGKIESIDMITDKGTGKKRGFCFITFEDYDTVDKLVLKKYLDFKGK     175
squid_prot2          DDTAEDDVREVFGRFGKIEKLEMIKDKNTGKQRGFCFITFDDFDCVDKCVLKRRISLNGK     178
                     :** *..:*: *   **** ::::.*:.:**:***.*:**:*.* *** *:::   .:*:

human_HNRNPA1        NCEVRKALSKQEMASASSSQRGRSGSGNFGGGRGGGFGGNDNFGRG--------------     219
human_HNRNPA1B       NCEVRKALSKQEMASASSSQRGRSGSGNFGGGRGGGFGGNDNFGRG--------------     219
squid_prot1          RVEVRKALSRAEMNHNKQISMGP-----MGGPMGPGPMGPGPMGPGPHMGGPMGPPHMGP     230
squid_prot2          FVEVKKAVSKDRDG--------G-----MGGGR---------GGRGG-------------     203
                       **:**:*: .                :**             * *
                                RGG
human_HNRNPA1        ---GNFSGRGGFGGSRGGGGYGGSG----DGYN---------------GFGNDG------     251
human_HNRNPA1B       ---GNFSGRGGFGGSRGGGGYGGSG----DGYN---------------GFGNDGGYGGGG     257
squid_prot1          PGPPGRGGRGGMGGGRGGDWHNSPGN---HGYG----GGGNFNGGYHQGPGN-WGGRGGH     282
squid_prot2          -SRGGMGGRGGFGGANGGGYNDNYGYQGNMGYQGNMGGGGGPQGGYGGGGGPQGYGGGN     262
                         . .****:**..**.   .. *     **                * *.
                                          7B
human_HNRNPA1        ------------------------------------------------SNFGGGGSYNDF     263
human_HNRNPA1B       PGYSGGS--RGYGSGGQGYGNQGSGYGGSGSYDSYNNGGGGGFGGGSGSNFGGGGSYNDF     315
squid_prot1          PGYGGYGHQGNYGGGHGGYGNQHGGYGGGGG----------GYGQ---G--GWNQGYEGF     327
squid_prot2          QGYGGGGGYGNSGGM-------GGRYGGPGG---------GGGSG---G--DFNDGYNNF     301
                                                                .   .  .  .*:.*
                                   M9
human_HNRNPA1        GNYNNQSSNFGPMKGGNFGGRSSGPYGGGGQYFAKPRNQGGYGGSSSSSSYGSGRRF   320
human_HNRNPA1B       GNYNNQSSNFGPMKGGNFGGRSSGPYGGGGQYFAKPRNQGGYGGSSSSSSYGSGRRF   372
squid_prot1          GDSG-----------------------------------GYGGGGGSGYSGGYRR-   347
squid_prot2          GSGYGSTYGGGPTRGASFAQRGAGPYGSGYGS--------GGGGGGGGGMGGYRR-   349
                     *.                                     * **.....  *. **
```

Figure S2

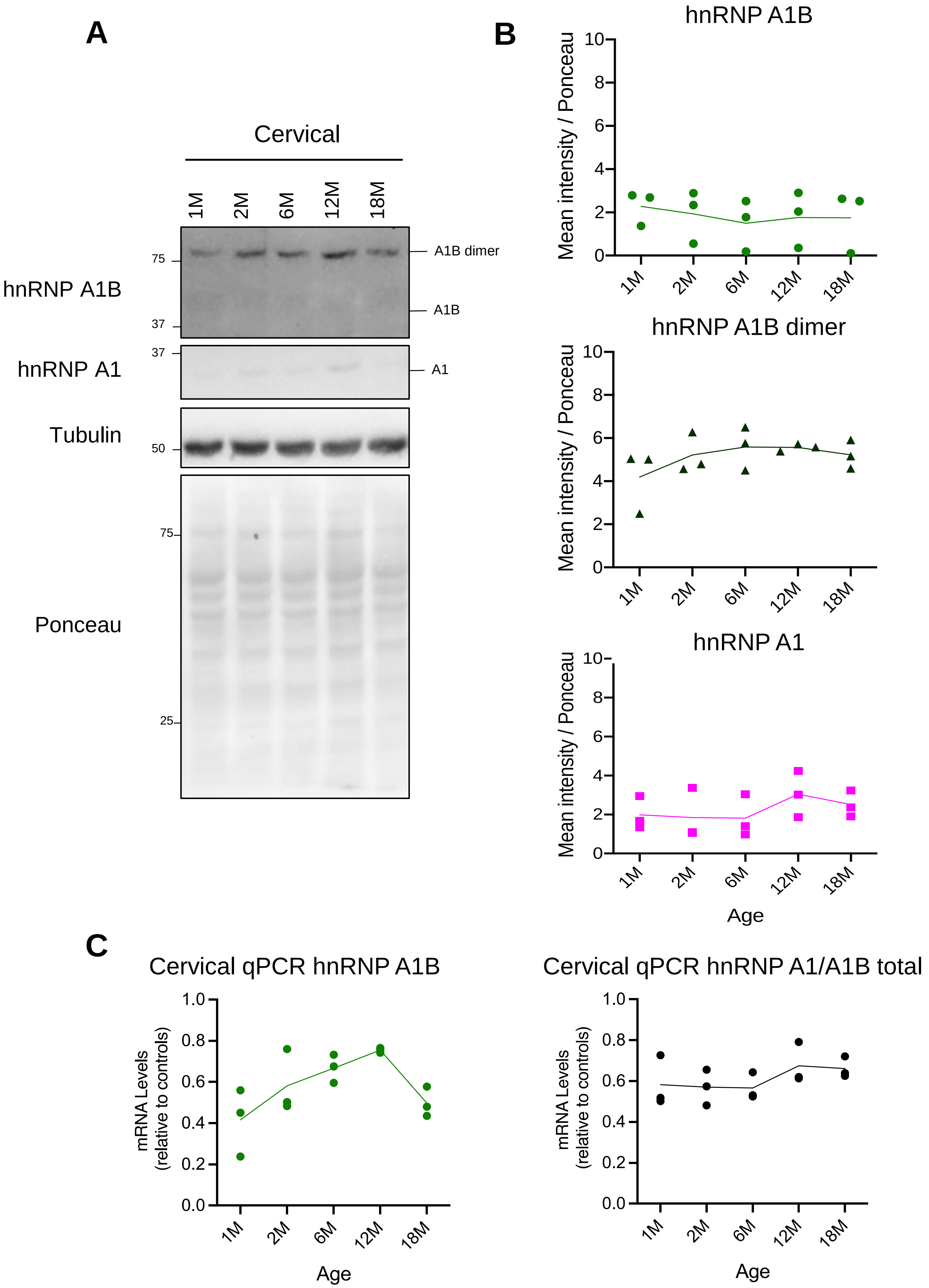
A
Cervical
1M
2M
6M
12M
18M
hnRNP A1B
75
37
A1B dimer
A1B
hnRNP A1
37
A1
Tubulin
50
Ponceau
75
25
B
hnRNP A1B
Mean intensity / Ponceau
10
8
6
4
2
0
1M
2M
6M
12M
18M
hnRNP A1B dimer
Mean intensity / Ponceau
10
8
6
4
2
0
1M
2M
6M
12M
18M
hnRNP A1
Mean intensity / Ponceau
10
8
6
4
2
0
1M
2M
6M
12M
18M
Age
C
Cervical qPCR hnRNP A1B
mRNA Levels
(relative to controls)
1.0
0.8
0.6
0.4
0.2
0.0
1M
2M
6M
12M
18M
Age
Cervical qPCR hnRNP A1/A1B total
mRNA Levels
(relative to controls)
1.0
0.8
0.6
0.4
0.2
0.0
1M
2M
6M
12M
18M
Age

Figure S3

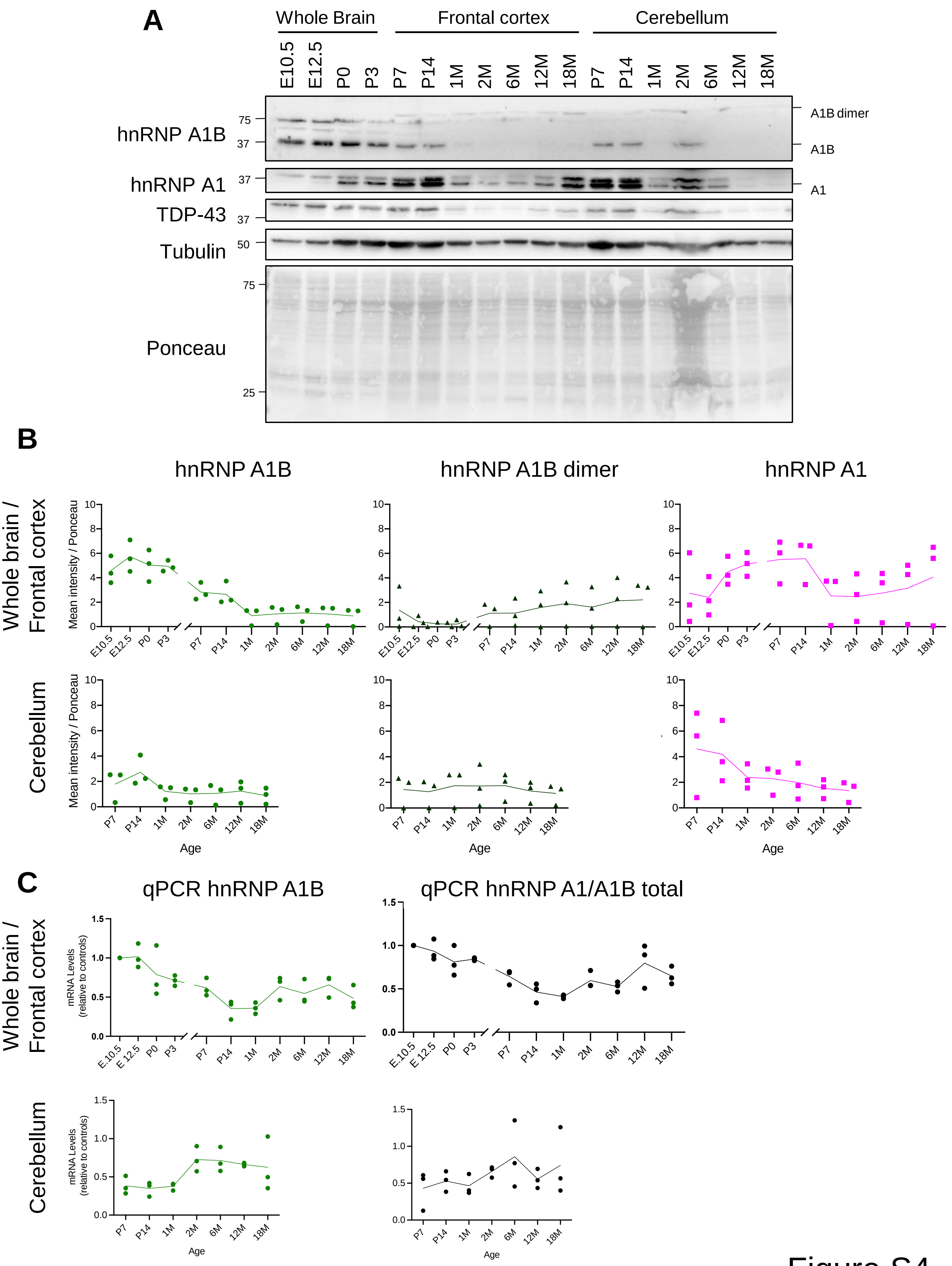
A
Whole Brain
Frontal cortex
Cerebellum
E10.5
E12.5
P0
P3
P7
P14
1M
2M
6M
12M
18M
hnRNP A1B
hnRNP A1
TDP-43
Tubulin
Ponceau
A1B dimer
A1B
A1
75
37
50
25
B
hnRNP A1B
hnRNP A1B dimer
hnRNP A1
Whole brain / Frontal cortex
Cerebellum
Mean intensity / Ponceau
Age
C
qPCR hnRNP A1B
qPCR hnRNP A1/A1B total
mRNA Levels (relative to controls)

Figure S4

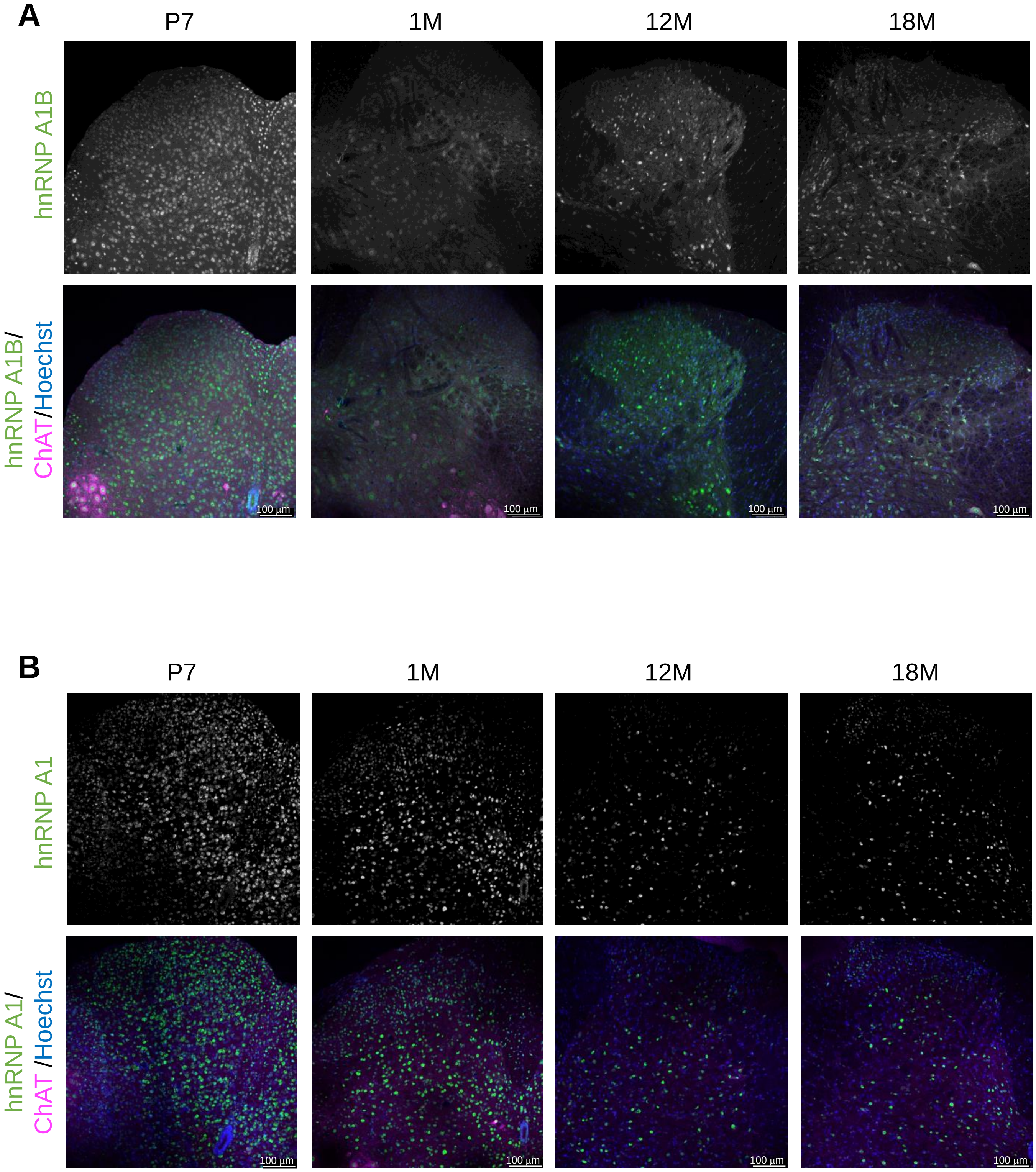
A
P7
1M
12M
18M
hnRNP A1B
hnRNP A1B/ ChAT/Hoechst
100 µm
B
P7
1M
12M
18M
hnRNP A1
hnRNP A1/ ChAT /Hoechst
100 µm

Figure S5

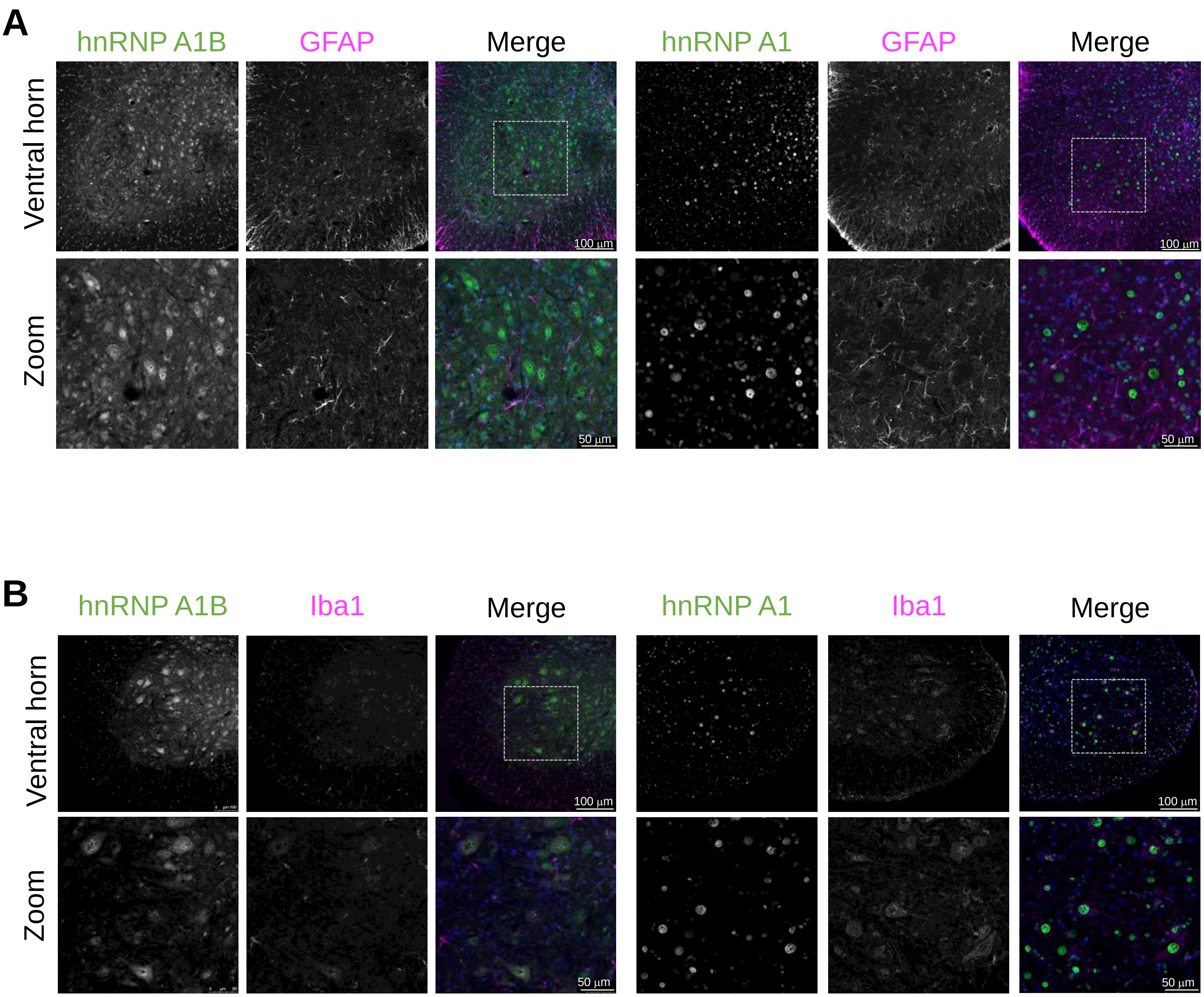
A
hnRNP A1B
GFAP
Merge
hnRNP A1
GFAP
Merge
Ventral horn
Zoom
100 µm
50 µm
B
hnRNP A1B
Iba1
Merge
hnRNP A1
Iba1
Merge
Ventral horn
Zoom
100 µm
50 µm

Figure S6

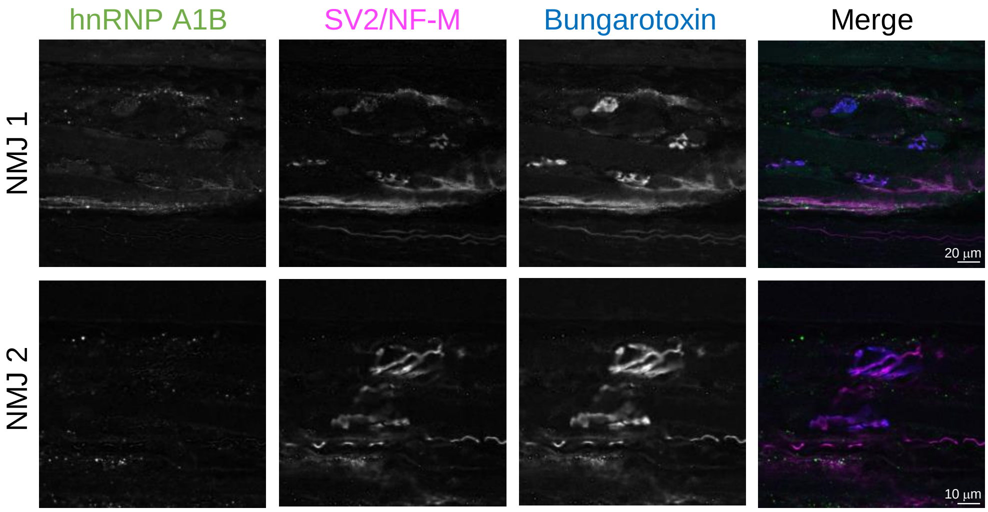

Figure S7
